# Full-term live birth in a woman with 17α-hydroxylase and 17,20-lyase deficiency with assisted reproductive technology: a case report

**DOI:** 10.1186/s12905-023-02492-z

**Published:** 2023-08-04

**Authors:** Sisi Xi, Xiuli Yang, Xuemin Shan, Qing Xue

**Affiliations:** Peking University First Hospital Obstetric and Gynecology Department Reproductive and Genetic Center, No.1 Xi’an Men Street, Xi Cheng District, Beijing, China

**Keywords:** 17α-hydroxylase deficiency, 17,20-lyase deficiency, CYP17A1 gene mutation, In vitro fertilization, Case report

## Abstract

**Background:**

17α-hydroxylase deficiency, which is caused by a CYP17A1 gene mutation, is a rare type of congenital adrenocortical hyperplasia that mainly manifests as hypertension, hypokalaemia and sexual dysplasia. To date, few pregnancies associated with this syndrome have been reported.

**Case presentation:**

We describe a 35-year-old Chinese woman with nonclassical congenital adrenal hyperplasia (NCCAH) due to 17α-hydroxylase/17,20-lyase deficiency who achieved pregnancy after in vitro fertilization (IVF) and frozen-thawed embryo transfer. She had secondary amenorrhea since she was 27, and subsequently, high level of progesterone in the follicular phase was found during a blood test. A compound heterozygous mutation was found in the CYP17A1 gene, c.1263G > A and c.985_987delinsAA. The patient was given standardized treatment with dexamethasone. Due to ovulation disorder, IVF was performed. She underwent whole embryo vitrification freezing. Frozen-thawed embryo transplantation was performed following the artificial cycle protocol of endometrium preparation, resulting in a singleton pregnancy. At 39 weeks and 1 day of gestation, caesarean section was performed due to the breech position of the foetus.

**Conclusion:**

A high level of progesterone reduces endometrial receptivity. Standardized treatment with dexamethasone and frozen-thawed embryo transfer with an artificial cycle protocol of endometrium preparation should be the choice for infertile female patients with CYP17A1 deficiency.

## Background

Congenital adrenal hyperplasia (CAH) is an autosomal recessive disorder resulting from the deficiency of enzymes required for adrenal cortical hormone production. Up to 90% of CAH cases are related to impaired steroidogenesis which are caused by 21α-hydroxylase deficiencies [1]. 17α-hydroxylase/17,20-lyase deficiency, which is caused by a CYP17A1 gene mutation, accounts for approximately 1% of cases and is a rare type of congenital adrenocortical hyperplasia [2]. The main clinical manifestations of CYP17A1 gene mutations are hypertension, hypokalaemia and sexual dysplasia. CYP17A1 regulates the synthesis of 17α-hydroxylase and 17,20-lyase and further adjusts the secretion of steroids in the gonads and cortisol in the adrenal glands. The clinical manifestations vary in different patients due to different degrees of activity of the above two enzymes. Irregular menstruation and infertility are the common causes for clinical visits in NCCAH cases. Spontaneous menarche of female patients suggests that the activity of 17α-hydroxylase and 17,20-lyase may be partially impaired. We report a full-term live singleton birth from a woman with partial 17α-hydroxylase/17,20-lyase deficiency who presented with secondary amenorrhea and infertility caused by compound heterozygous CYP17A1 mutations. The study was approved by the ethics committee of Peking University First Hospital, and the patient provided written informed consent.

## Case presentation

The patient’s menarche began when she was 14 years old. Thereafter, menses were irregular (intervals of 3 to 6 months between periods) and lasted 3 days. Her menstrual cycle had extended to eight to twelve months when she was 25 years old. She married at the age of 26. She had secondary amenorrhea since she was 27, and she needed to intermittently take progesterone to induce menstruation. When she had suffered from primary infertility for 5 years, she came to the Reproductive Medicine Center of Peking University First Hospital to seek treatment. She had normal external female genitalia. Pelvic ultrasound examination revealed a normal uterus. Her blood pressure and serum potassium level were normal during the examination. The semen analysis of her husband revealed asthenozoospermia (concentration: 76 × 10^6^ sperm cells /ml; percentage of progressive motility: 29%; percentage of normal forms sperm cells: 6.4%). The female patient received three courses of ovulation induction therapy and artificial insemination but did not conceive. Sex hormone tests showed that the serum progesterone level was 9.08 ng/ml in the early follicular phase, and the serum 17-hydroxyprogesterone (17OHP) level was 2.42 ng/ml. The basal levels of FSH, LH, oestradiol, testosterone and prolactin were 6.65 mIU/ml, 7.41 mIU/ml, 19.00 pg/ml, 0.31 ng/ml and 16.47 ng/ml, respectively. The level of thyroid stimulating hormone(TSH) was 2.51μIU/ml. It was inferred that she may suffer from congenital adrenal hyperplasia, and she was referred to the endocrinology department for further diagnosis and treatment.

The adrenocorticotropic hormone (ACTH), cortisol and other related hormone levels are shown in Table 1. The patient underwent a mid-dose dexamethasone suppression test, and the results are shown in Table 2.

**Table 1:** Baseline hormone levels

Hormones	Value	Reference range	Unit
ACTH	108.0	0–46	pg/ml
Cortisol	117.3	40–223	ng/ml
17α-hydroxypregnenolone	4.81	0.31–4.55	ng/ml
Androstenedione	0.337	0.35–5.20	ng/ml

**Table 2:** Baseline hormone levels and responses to mid-dose dexamethasone suppression test

Hormones	Basal	Post-therapy	Reference range for basal levels	Unit
ACTH	68.2	< 5	0–46	pg/ml
17OHP	1.95	0.55	0.1–0.8	ng/ml
Progesterone	4.91	0.52	0.38–2.28	ng/ml
Testosterone	0.13	< 0.1	0.10–0.75	ng/ml
Dehydroepiandro-steronesulfate	128.9	75.5	23–266	μg/dl

Her karyotype was 46, XX. A mid-dose dexamethasone suppression test revealed that the levels of ACTH, 17OHP and progesterone could be inhibited. A CT scan showed thickening of the medial limb of the left adrenal gland. CYP17A1 gene mutation analyses were performed for this patient, her parents and her husband. A compound heterozygous mutation was found in the CYP17A1 gene (NM_000102): c.1263G > A (p.A421A) and c.985_987delinsAA (p.Y329Kfs*90) in this female patient. The sequencing results of her parents showed that her mother was a heterozygous carrier for c.1263G > A, and her father was a heterozygous carrier for c.985_987delinsAA. No mutation was found in the CYP17A1 gene in her husband. She was diagnosed with NCCAH-17α hydroxylase deficiency and was treated with dexamethasone 0.75 mg once a day; this dosage was reduced to 0.375 mg once a day after the progesterone level returned to normal.

She returned to the reproductive medicine center at 35 years of age and was willing to continue artificial pregnancy treatment. After treatment with oral glucocorticoids, her serum progesterone level dropped to 1.6 ng/ml in the early follicular phase. The antral follicle count (AFC) of the bilateral ovaries was 11 at menstrual cycle day 3, and the anti-Mullerian hormone (AMH) value was 2.85 ng/ml. The basal levels of FSH, LH and oestradiol were 11.5 mIU/ml, 7.6 mIU/ml and 18 pg/ml, respectively. The level of TSH was 3.92μIU/ml. At the first IVF cycle, ovarian stimulation was conducted with recombinant FSHα (rFSH) and hMG, and the initial dose of gonadotropin was 375 IU. After 9 days of ovarian hyperstimulation with 3450 IU gonadotropins in total, there were 8 follicles with diameters larger than 14 mm, and serum oestradiol was 156 pg/mL. The progesterone levels ranged from 1.60 to 8.24 ng/ml during the ovarian stimulation treatment. No GnRH antagonist or GnRH agonist was used in this cycle. Recombinant human chorionic gonadotropin was used as the final maturation trigger. Four mature oocytes were obtained, and 3 transplantable embryos (grade III) formed on the third day after oocyte retrieval and fertilization with IVF technology. One transplantable cleavage embryo was cryopreserved by vitrification. The other 2 cleavage embryos were cultured sequentially to the blastocyst stage, but no transplantable blastocysts formed. At the second IVF cycle, similar ovarian stimulation drugs were used, and the initial dose of gonadotropin was 375 IU. After 6 days of ovarian hyperstimulation with 2400 IU gonadotropins, there were 5 follicles with diameters larger than 14 mm, and the serum oestradiol level was 163 pg/mL on the trigger day. The progesterone levels ranged from 0.47 to 4.93 ng/ml during the ovarian stimulation treatment. Three mature oocytes were obtained. One transplantable cleavage embryo and 1 transplantable blastocyst were cryopreserved by vitrification. Two months after the second oocyte retrieval operation, her serum progesterone level was 0.57 ng/mL after treatment with oral dexamethasone (0.375 mg/d).

The artificial cycle protocol of endometrium preparation was selected, and she began taking oral oestradiol valerate 3 mg three times a day. When the endometrial thickness reached 9 mm after 14 days of oestradiol treatment, progesterone intramuscular injection (60 mg/d) and dydrogesterone tablets (10 mg three times a day) were administered for endometrial transformation. She conceived a singleton pregnancy after the first frozen embryo transfer attempt with 2 cleavage-stage embryos. There were no complications during pregnancy, and the foetus developed normally. At 39 weeks and 1 day of gestation, caesarean section was performed due to the breech position of the foetus. A live female newborn with a birth weight of 3490 g and length of 50 cm was delivered. The Apgar scores of the newborn were 10/10/10. She recovered well after the surgery and was discharged as scheduled. The female newborn was discharged with her mother. The baby is now 1 year old and is developing normally.

## Discussion and conclusions

CAH may be caused by homozygous gene mutations or compound heterozygous gene mutations. The clinical phenotype depends on the activity of residual enzymes. To date, few pregnancies associated with this syndrome have been reported. In this study, we report a female patient with compound heterozygous CYP17A1 mutations, with a frame shift mutation c.985_987delinsAA in one allele and a synonymous substitution (c.1263G > A) in the other allele. The c.985_987delinsAA mutation leads to truncation of protein synthesis at codon 417, thus abolishing CYP17A1 activity [3]. Since synonymous mutations only affect DNA and mRNA but not the encoded amino acids, they have been viewed as silent mutations. Recent studies have revealed their functions in the structure or function of mRNA [4, 5]. As the c.1263G > A mutation of CYP17A1 is a synonymous substitution, the codons before and after the mutation induce the synthesis of the same amino acid. Qiao et al. [6] showed that the c.1263G > A mutation of CYP17A1 leads to aberrant splicing of mRNA and a missing portion of exon 8, thus inducing a deletion of 6 or 7 amino acids starting from residue position 415. This may be the reason why the compound heterozygous CYP17A1 mutation led to 17α-hydroxylase/17,20-lyase deficiency in this patient.

Successful live births have been reported in 4 patients with combined 17α-hydroxylase and 17,20-lyase deficiency [7–10], while only 1 patient with isolated 17,20-lyase deficiency was reported to have delivered [11]. Blood tests of the patient in this report showed elevated 17α-hydroxypregnenolone and 17OHP levels, which revealed the residual function of 17α-hydroxylase/17,20-lyase. However, the low levels of androstenedione and oestrogen revealed a potential deficiency of 17,20-lyase. Based on the CYP17A1 genetic analyses, we concluded that this patient was suffering mainly from 17,20-lyase deficiency. The 17α-hydroxylase activity in this patient was partially preserved; thus, she did not suffer from hypokalaemia or hypertension. The high level of progesterone during ovulation induction did not impair the developmental potential or the quality of oocytes, but the unusually high level of progesterone and low level of oestradiol influenced the thickness and morphology of the endometrium, eventually reducing endometrial receptivity. The controlled ovarian hyperstimulation protocol for this patient was approximate to the ovulation induction protocol of the natural luteal phase. There was no need to use GnRH antagonists or GnRH agonists during the gonadotropin stimulation period, as the high level of serum progesterone throughout the cycle inhibited ovulation. Subsequent artificial endometrial preparation and frozen embryo transfer are effective infertility treatments for women with CYP17A1 deficiency.

In conclusion, this is a success case of a woman with 17α-hydroxylase/17,20-lyase deficiency caused by compound heterozygous mutations of CYP17A1 who achieved live birth by IVF and frozen embryo transplant. The high level of progesterone and low level of oestradiol reduced endometrial receptivity. Standardized treatment with dexamethasone and frozen-thawed embryo transfer with an artificial cycle protocol of endometrium preparation should be the choice for infertile female patients with CYP17A1 deficiency.

## Data Availability

The datasets used and/or analysed during the current study available from the corresponding author on reasonable request.

## Abbreviations

CAH: Congenital adrenal hyperplasia
NCCAH: Non-classical congenital adrenal hyperplasia
IVF: In vitro fertilization
GnRH: Gonadotropin releasing hormone
17OHP: 17-Hydroxyprogesterone
ACTH: Adrenocorticotropic hormone
FSH: Follicle-stimulating hormone
LH: Luteinizing hormone
hMG: Human menopausal gonadotropin
CT: Computed tomography
AFC: Antral follicle count
AMH: Anti-Mullerian hormone
DNA: Deoxyribonucleic acid
mRNA: Messenger ribonucleic acid
TSH: Thyroid stimulating hormone
